# Anorectal Complications During Neutropenic Period in Patients with Hematologic Diseases

**DOI:** 10.4084/MJHID.2016.019

**Published:** 2016-03-01

**Authors:** Soner Solmaz, Aslı Korur, Çiğdem Gereklioğlu, Süheyl Asma, Nurhilal Büyükkurt, Mutlu Kasar, Mahmut Yeral, İlknur Kozanoğlu, Can Boğa, Hakan Ozdoğu

**Affiliations:** 1 Department of Hematology, Adana Hospital of Başkent University, Adana, Turkey; 2 Department of Family Medicine, Adana Hospital of Başkent University, Adana, Turkey; 3 Department of Hematology Research Laboratory, Adana Hospital of Başkent University, Adana, Turkey

## Abstract

**Background:**

Neutropenic patients are susceptible to any anorectal disease, and symptomatic anorectal disease afflicts 2–32% of oncology patients. Perianal infections are the most feared complication, considering the lack of natural defense against infectious microorganisms. When septic complications develop, the anorectal disease is potentially fatal, especially in neutropenic patients in whom mortality rates range between 11–57%. Although anorectal diseases are a frequent complication with potentially fatal outcomes among patients with hematologic diseases, sufficient data are not available in the literature. In this study, we aimed to investigate the anorectal complications developing during the neutropenic period in patients with hematologic diseases.

**Methods:**

A total of 79 patients whose neutropenic period (absolute neutrophil count <500/mcL) continued for 7 days, or longer were included in the study.

**Results:**

A total of 34 patients out of 79 (43%) were detected to develop anorectal complications, of them 6 (7.6%) developed an anorectal infection. The patients were characterized according to the hematological disease and its status (active or not), the type of treatment and the presence of a history of an anorectal pathology before the onset of the hematologic disease. Nineteen (24.1%) patients had the history of anorectal disturbances before diagnosis of the hematologic disease, and recurrence of an anorectal pathology was found in 14 out of 19 patients(73.7%). In addition, the overall mortality rate was higher among the patients who developed anorectal complications compared to another group (41.2% vs. 22.2%, p=0.059).

**Conclusion:**

Anorectal pathology is a common complication with high recurrence rate in neutropenic patients. Perianal infections are important as they can cause life-threatening outcomes although they are relatively rare among all anorectal complications. Therefore perianal signs and symptoms should be meticulously evaluated concerning early diagnosis and treatment.

## Introduction

Febrile neutropenia is an important cause of mortality occurring commonly after myelosuppressive treatment in cancer patients.^1^,^2^ Neutropenic patients are susceptible to any anorectal complications,^3^ and symptomatic anorectal pathology afflicts 2–32% of oncology patients.^4^ Perianal infections (represented by an abscess or infected fistula) are the most feared complication, considering the lack of natural defense against infectious microorganisms.^3^ The incidence of perianal infection is approximately 8–9% of patients with acute leukemia.^5^ When septic complications develop, the anorectal disease is potentially fatal, especially in neutropenic patients, in whom mortality rates range from 11–57%.^4^

Although anorectal diseases are a frequent complication with potentially fatal outcomes among patients with hematologic diseases, sufficient data are not available in the literature. In this study, we aimed to investigate the anorectal complications developing during the neutropenic period in patients with hematologic diseases.

## Methods and Patients

This study was conducted as a retrospective, cross-sectional, single-center investigation. A total of 79 patients, admitted to our clinic between 01 November 2014 and 01 November 2015, with a diagnosis of acute myeloid leukemia (AML), acute lymphoblastic leukemia (ALL), myelodysplastic syndrome (MDS), Hodgkin lymphoma (HL), non-Hodgkin lymphoma (NHL), multiple myeloma (MM) and aplastic anemia (AA), whose neutropenic period (absolute neutrophil count <500/mcL) continued for 7 days or longer, were included in the study. Anorectal problems were defined according to anamnesis, physical examination findings, gastroenterology and general surgery consultations, and imaging methods (pelvic computer tomography or magnetic resonance imaging) when required.

Patients who had a newly diagnosed disease, or refractory, relapsed and progressive were defined as the ones who had “an active disease”. Comorbidities included diabetes mellitus, hypertension, a pulmonary, cardiac, hepatic and renal disease, or a history of a previous malignancy other than the current one,

### Statistical Analysis

Patient characteristics were examined using descriptive statistics. Continuous variables were given as mean ± standard deviation (SD), and categorical variables were defined as a percentage. Chi-square test and t-test were used to compare proportions and means for categorical and continuous variables, respectively. Statistical Significance was defined as p<0.05. All test significances were two-tailed. SPSS statistical software (SPSS 17.0 for Windows, Inc., Chicago, IL, USA) was used for all statistical calculations.

## Results

The study included 30 (38%) female and 49 (62%) male patients with mean age of 42.9±14.4 years (44.8±15.6 for women and 41.8±13.7 for men). Clinical characteristics of the patients are given in Table 1.

The mean duration of neutropenia period was found as 14.6 days (range 7–60). A total of 34 patients out of 79 (43%) were detected to develop anorectal complications, of them 6 (7.6%) developed a perianal infection (abscess and fistula). Anorectal complications were observed on average by day 12 (0–28) of neutropenia, and the period of neutropenia lasted an average of 8 days (range 1–60). When the patients were compared regarding anorectal complications, the patients who developed anorectal complication were seen to be older (43.8±14.7 vs. 42.3±14.4 years, p=0.661) and stayed neutropenic for a longer period (16.7±7.5 vs. 13.7±8.2 days, p=0.102) however the difference was not statistically significant for both parameters. Comparison of the groups about anorectal complications development is given in Table 2.

Of 6 patients who developed a perianal infection, septic shock develop in 3, Fournier’s gangrene developed in one, and the overall mortality rate was 50%. Culture positivity was detected in 2 (33.3%) out of 6 patients, and growing microorganisms were Pseudomonas aeruginosa and Escherichia coli.

An anorectal complication was seen to develop in a total of 34 (43%) patients during the neutropenic period. Nineteen patients ((24.1%) had the history of anorectal diseases before the diagnosis of the hematologic disease, and a recurrence of anorectal disease was found in 14 out of 19 patients ( 73.7%). Of 60 patients without previous anorectal diseases 20 (33,3 %) suffered from an anorectal complication, and a statistically significant difference was detected between the two groups (p=0.003).). The anorectal complication was detected in 29 out of 53 patients (54. 7%) who had acute leukemia and MDS, in 5 out of 23 patients (21.7%) who had lymphoma and MM, and no anorectal complications developed in AA patients. Febrile neutropenia ratio was higher among the patients who developed anorectal complications as expected although the difference was not significant (82.4% vs. 73.3%, p=0.344).

When the groups were compared, the disease type (acute leukemia or MDS vs. lymphoma), the presence of active disease, the kind of treatment and presence of the history of an anorectal pathology before the diagnosis of the hematologic disease were influent in determining anorectal disease development. On the other hand, corticosteroid use was detected not to be effective on anorectal complication development. A statistically significant difference was not detected between the groups which developed and did not develop anorectal complications in steroid use (41.2% vs. 31.1%, respectively; p=0.354). In addition, the overall mortality rate was higher among the patients who developed anorectal complications compared to another group (41.2% vs. 22.2%, p=0.059). In our study, 14 out of 23 patients (60.9%) who developed anorectal complications and had an active disease died, however, no deaths occurred in 11 patients who developed anorectal complications but who did not have an active disease.

## Discussion

Manifestations of perianal infections may differ from those with a competent immune system^3^ and are often accompanied by severe pain, swelling, constipation, and may cause systemic infection.^6^ However, the clinical presentation of an anorectal infection is often masked by the absence of inflammatory cells so recognition of the signs and symptoms can be difficult in neutropenic patients.^7^ Perianal infections are a life-threatening complication including Fournier’s gangrene that requires rapid diagnosis and intervention as recurrence and mortality rates may be expressive.^3^,^8^ In a series of 92 patients with acute or chronic leukemia, most common manifestations were a perirectal abscess (27%) followed by anal fissures (23%), external hemorrhoids (19%) and perianal ulcerations (13%).^3^ Another retrospective study found a perianal infection prevalence of 6.7% and recurrence was diagnosed in 31% of the cases.^3^ Grewal et al.^4^ reported that 5.8% patients hospitalized with leukemia had the concomitant symptomatic anorectal disease. Büyükaşık et al.^5^ found the incidence of perianal infections in acute leukemia as 7.3 percent. We detected that 43% of the patients developed anorectal complications, and 7.6% developed a perianal infection (abscess and fistula), consistently with literature. Recurrence of anorectal disease was found as 73.7% (14 out of 19 patients) in our study suggesting that anorectal disease development is a common and recurrent complication during the neutropenic period. Anorectal complication development rate was higher among the patients who had an active disease compared to the patients whose disease was under control (67.6% vs. 40.0, p=0.015). This finding suggests that anorectal complications can cause severe morbidity in the presence of an active disease. While overall mortality rate was found in 41.2% of all patients with anorectal complication, this proportion increases to 60.9% in the presence of active disease together. On the other hand, no deaths occurred in 11 patients who had an anorectal complication but not an active disease. Similarly to our results, Musa et al.^9^ found that the overall mortality was 53% among 17 adults with hematologic malignancy in whom anorectal complications developed, the death rate was 69% for those in whom the disease was not in remission compared with zero for patients who were in remission. However, these data are insufficient for to say that anorectal complications increase mortality in patients with active disease.

In literature, no consensus is available for the treatment of anorectal complications in neutropenic patients together with the lack of studies investigating these complications.^5^ Some authors defended operative treatment, whereas others reported high mortality rate with operative treatment compared with medical procedures.^5^ Interestingly, internists reported success with surgery or failure with medical treatment; however, most of the surgeons were unsatisfied with the surgical approach and concluded that surgical treatment should be reserved for patients who recovered from neutropenia and active disease.^5^ Grewal et al.^4^ did not observe excessive morbidity or mortality in the operated neutropenic patients with anorectal disease as compared with the non-operated patients. Nowadays, the number of patients requiring surgical intervention decreased substantially, which it was ascribed to the use of broad-spectrum antibiotics coverage of gram-negative and anaerobic bacteria.^7^ We had a similar clinical approach; namely, we prefer administration of a broad spectrum antibiotic effective on gram negative and anaerobic bacteria (usually a carbapenem antibiotic) when perianal infection develops during the severe neutropenic period. The surgical approach is considered in the case of failure of medical treatment or persistence of the perianal infection even after the restore from neutropenia.

## Conclusion

An anorectal pathology is a common complication with high recurrence rate in neutropenic patients. It is a serious cause of morbidity which impairs quality of life particularly in patients with acute leukemia and MDS, who receive intensive chemotherapy and who have an active disease. Perianal infections are important as they can cause life-threatening outcomes although they are relatively rare among all anorectal complications. Therefore perianal signs and symptoms should be meticulously evaluated to do an early diagnosis and treatment.

## Figures and Tables

**Table 1:** Clinical characteristics of the patients.

	n	%

Gender		
• Male	49	62.0
• Female	30	38.0

Diagnosis		
• Acute myeloid leukemia	34	43.0
• Acute lymphoblastic leukemia	14	17.7
• Lymphoma	13	16.5
• Multiple myeloma	10	12.7
• Myelodysplastic syndrome	5	6.3
• Aplastic Anemia	3	3.8

Number of comorbidity		
• No	57	72.2
• 1	19	24.1
• 2	3	3.8

Disease status		
• Active Disease	41	51.9
• No Active Disease	38	48.1

ECOG performance status		
• 0	22	27.8
• 1	44	55.7
• 2	8	10.1
• 3	5	6.3

Type of treatment		
• Chemotherapy	43	54.4
• Hematopoietic stem cell transplantation	35	44.3
• Other	1	1.3

Use of Corticosteroid	28	35.4

Neutropenic fever		
• No	18	22.8
• Yes	61	77.2

History of anorectal disease before diagnosis of the hematologic disease		
• No	60	75.9
• Internal/external hemorrhoidal disease	13	16.5
• Fissure with or without hemorrhoid	5	6.3
• Fistula	1	1.3
• Abscess	0	0

Anorectal complication developing in the course of neutropenic period		
• No	45	57.0
• Symptomatic internal/external hemorrhoid	8	10.1
• Fissure with or without hemorrhoid	20	25.3
• Fistula	2	2.5
• Abscess	4	5.1

**Table 2:** Comparison of the groups with regard to anorectal complication development.

	Anorectal complication		P value
	Yes (n=34)	No (n=45)	

Gender			0.670
• Male	22 (64.7%)	27 (60.0%)	
• Female	12 (35.3%)	18 (40.0%)	

Diagnosis			0.009
• Acute leukemia and myelodysplastic syndrome	29 (85.3%)	24 (53.3%)	
• Lymphoma and multiple myeloma	5 (14.7%)	18 (40.0%)	
• Aplastic anemia	0	3 (6.7%)	

Number of comorbidity			0.753
• No	26 (76.5%)	31 (68.9%)	
• 1	7 (20.6%)	12 (26.7%)	
• 2	1 (2.9%)	2 (4.4%)	

Active disease			0.015
• Yes	23 (67.6%)	18 (40.0%)	
• No	11 (32.4%)	27 (60.0%)	

ECOG performance status			0.766
• 0	8 (23.5%)	14 (31.1%)	
• 1	19 (55.9%)	25 (55.6%)	
• 2	4 (11.8%)	4 (8.9%)	
• 3	3 (8.8%)	2 (4.4)	

Type of treatment			0.0001
• Chemotherapy	30 (88.2%)	13 (28.9%)	
• Hematopoietic stem cell transplantation	3 (8.8%)	32 (71.1%)	
• Other	1 (2.9%)	0	

Febrile neutropenia			0.344
• No	6 (17.6%)	12 (26.7%)	
• Yes	28 (82.4%)	33 (73.3%)	

**Use of corticosteroid**	**14 (41.2%)**	**14 (31.1%)**	**0.354**

History of anorectal disease before diagnosis of the hematologic disease			0.005
• No	20 (58.8%)	40 (88.9%)	
• Yes	14 (41.2%)	5 (11.1%)	

Death			0.059
• No	20 (58.8%)	35 (77.8%)	
• Yes	14 (41.2%)	10 (22.2%)	
